# Quantitative Analysis of Food and Feed Samples with Droplet Digital PCR

**DOI:** 10.1371/journal.pone.0062583

**Published:** 2013-05-02

**Authors:** Dany Morisset, Dejan Štebih, Mojca Milavec, Kristina Gruden, Jana Žel

**Affiliations:** Department of Biotechnology and Systems Biology, National Institute of Biology, Ljubljana, Slovenia; Lawrence Berkeley National Laboratory, United States of America

## Abstract

In this study, the applicability of droplet digital PCR (ddPCR) for routine analysis in food and feed samples was demonstrated with the quantification of genetically modified organisms (GMOs). Real-time quantitative polymerase chain reaction (qPCR) is currently used for quantitative molecular analysis of the presence of GMOs in products. However, its use is limited for detecting and quantifying very small numbers of DNA targets, as in some complex food and feed matrices. Using ddPCR duplex assay, we have measured the absolute numbers of MON810 transgene and *hmg* maize reference gene copies in DNA samples. Key performance parameters of the assay were determined. The ddPCR system is shown to offer precise absolute and relative quantification of targets, without the need for calibration curves. The sensitivity (five target DNA copies) of the ddPCR assay compares well with those of individual qPCR assays and of the chamber digital PCR (cdPCR) approach. It offers a dynamic range over four orders of magnitude, greater than that of cdPCR. Moreover, when compared to qPCR, the ddPCR assay showed better repeatability at low target concentrations and a greater tolerance to inhibitors. Finally, ddPCR throughput and cost are advantageous relative to those of qPCR for routine GMO quantification. It is thus concluded that ddPCR technology can be applied for routine quantification of GMOs, or any other domain where quantitative analysis of food and feed samples is needed.

## Introduction

In many aspects of basic research, diagnostic tests, and commercial processes, the advent of modern analytical technologies has provided the ability to detect and quantify nucleic acid targets with unprecedented sensitivity and specificity. Currently, the most common technique for analyzing the presence of nucleic acids in food and feed samples is the polymerase chain reaction (PCR) [1]–[3]. When quantitative analysis is required, the use of real-time quantitative PCR (qPCR) is preferred because of its accuracy and precision [1]. However, its use for target quantification can be seriously limited by a significant bias when the target is present at low concentrations in a background of high numbers of non-target nucleic acids in the sample [4]–[7]. Another important limitation is its sensitivity to the frequent presence of inhibitors co-extracted with nucleic acid from complex matrices [8].

One example of the need for quantitative nucleic acid analysis in food and feed is the testing for genetically modified organisms (GMOs). Numerous countries have implemented regulations requiring the labeling of products containing GMOs, or materials derived from GMOs, above certain thresholds, therefore emphasizing the requirement for quantification of GMO content [9].

GMO content in food and feed samples is expressed in relative terms as the ratio of the quantity of the transgene (GM target, *i.e*. the nucleic acid fragment introduced in the host genome) to that of the endogene (reference gene in the host genome) [10]. Using qPCR–the common technique for GMO quantification-, standard curves for the quantities of endogene and transgene are prepared separately, using serial dilutions of DNA extracted from reference material [8]. qPCR efficiency and hence quantification by qPCR of endogene and transgene, can be influenced by many factors, including inhibitors, present in food and feed samples, leading to significant under- or over-estimation of GMO content [8], [11]. Much effort has been put into improving the performance of qPCR quantification with respect to the inhibition and matrix effects [8] and the low concentration levels of targets in routine samples [12], [13]. The lack of certified reference material has also been noted [13]. However, most of the proposed solutions are not practical and reliable quantification of GMOs in food and feed samples still remains a major challenge.

The basis of digital PCR (dPCR) is to quantify the absolute number of targets present in a sample, using limiting dilutions, PCR and Poisson statistics [14]. To do so, the PCR mix is distributed across a large number of partitions containing zero, one or more copies of the target nucleic acid. After end-point PCR amplification, each partition is scrutinized and defined as positive (“1”, presence of PCR product) or negative (“0”, absence of PCR product) hence the term “digital”. The absolute number of target nucleic acid molecules contained in the original sample before partitioning can be calculated directly from the ratio of positive to total partitions, using binomial Poisson statistics [15].

Currently, two approaches are used in commercially available dPCR systems [15], [16]. One approach, termed chamber digital PCR (cdPCR), relies on the partitioning of up to a few thousand individual reactions in microfluidic chambers. The second approach, called droplet digital PCR (ddPCR), combines partitioning of the PCR test into several thousands or millions of individual droplets in a water-oil emulsion, with the use of flow cytometry to count positive PCR tests.

dPCR has been adopted for a number of applications, including studies of copy number variation involving allelic discrimination or imbalance, single cell gene expression, hyper-methylation, detection of low copy number nucleic acid targets (reviewed [6], [17], [18]) and of point mutations. Recently, a cdPCR commercial system has been demonstrated that enables suitable metrological performance for the certification of the copy number ratio of reference materials used in GMO testing [11], [19]. Several advantages are proposed for the use of dPCR instead of qPCR in routine GMO testing: 1) it enables absolute target copy number to be detected and avoids the amplification efficiency bias observed with qPCR [11], [19], 2) it overcomes dependence on the availability of references or standards [18], 3) it provides data with the high precision and confidence necessary for metrological use [11], [19], 4) it provides more accurate data at low target copy numbers than qPCR [20], allowing quantification of low GMO content, and 5), because of its tolerance to inhibitors as an end-point measurement, it can reduce the biases linked to matrix type often observed with qPCR [18]. However, the application of cdPCR is limited by two important factors: the small dynamic range it offers (2–3 logs) and its relatively high price.

Given the larger number of replicates allowed by ddPCR than by cdPCR and the lower price per sample of the former, it has been envisaged that ddPCR could allow better precision [16], confidence and easier adoption of digital PCR technology in laboratories for daily analysis, all at lower cost per sample [15]. The aim of this study was therefore to evaluate the application of ddPCR for quantitative analysis in food and feed samples. Taking GMO testing as a concrete example, ddPCR key performance parameters, using the QX100 droplet system (Bio-Rad, Pleasanton, CA), were compared with current qPCR performance and with the recently studied performance of cdPCR [11], [18]. Linearity of response, absolute limits of detection and quantification, repeatability over the dynamic range of the ddPCR endogene and transgene assays were assessed. The applicability of ddPCR with different sample matrices and the practicability of use for routine GMO testing were also evaluated. The results obtained should be applicable to other fields where quantitative testing in food and feed samples is required.

## Materials and Methods

### Test material

Several MON-ØØ81Ø-6 (MON810) maize seed powder based, certified reference materials (CRM) were purchased from the EU Joint Research Centre, IRMM (Institute for Reference Materials and Measurements, Geel, Belgium). All these CRMs have certified mass/mass (m/m) GM maize/wild-type maize material ratios. Some of them are also certified for the copy/copy (cp/cp) transgene/endogene ratio (see Appendix S1 and Table S1).

Other samples containing the MON810 maize event, and previously assayed by qPCR, were also used in this study. Finally, a limited specificity study was conducted on two samples containing either wild-type maize, or DNA from a milk sample without maize (see Appendix S1 and Table S1).

### DNA extraction

DNA was extracted and purified from 200 mg of starting material for all samples using the NucleoSpin Food kit (Macherey-Nagel GmbH & Co. KG, Düren, Germany). In parallel, DNA from sample G147/08 was also extracted and purified according to a modified CTAB method (see Appendix S1) [21].

Dilutions of the stock extracted DNA solutions were made with nuclease- and protease-free water (Sigma-Aldrich Chemie Gmbh, Munich, Germany), using pipettes calibrated with a SAG285 precision weighing module (Mettler-Toledo d.o.o., Ljubljana, Slovenia). All samples were stored at −20°C.

### Enzymatic restriction of genomic DNA

Enzymatic digestion of MON810 genomic DNA (gDNA) with *Taq*I (New England Biolabs GmbH, Frankfurt am Main, Germany) was performed as described (see Appendix S1) [19]. 6 µL of digested gDNA were analyzed on a 1% agarose gel to confirm complete digestion.

### qPCR reactions and data analysis

The *hmg* gene was used as the endogenous control gene for maize. A unique, single copy DNA integration-border region of the genomic sequence and the inserted sequence element originating from CaMV (35S promoter) were used for specific detection and quantification of the MON810 event. Probe and primer nucleotide sequences were the same as in the inter-laboratory validated protocol [22] but the TAMRA quencher in the probes was replaced by the Black Hole Quencher 1 (BHQ-1). The same primers and probes were used for both qPCR and ddPCR experiments (see Appendix S1 and Table S2).

MON810 content was determined by qPCR, using relative quantification according to the standard curve approach. Standard curves were prepared from five serial dilutions of the copy/copy ratio certified reference material ERM-BF413gk (starting from approximately 100 ng to 1 ng DNA per reaction) and used in two replicates. For each sample, the quantification was done based on two replicates of three dilutions. Results of quantification performed with CRM certified for transgene/endogene copy ratio were expressed as percentages of the copy/copy ratio.

### Droplet Digital PCR reactions and data analysis

Duplex ddPCR reaction mixes were prepared as follows. 10 µL of 2× ddPCR Master Mix (Bio-Rad, Pleasanton, CA) and 1 µL of each primer (final concentration of 300 nM) and probe (final concentration of 180 nM) were mixed, and 4 µL of DNA template added. For singleplex reactions, 3 µL of nuclease- and protease-free water (Sigma-Aldrich Chemie Gmbh, Munich, Germany) were added to complete a 20 µL reaction volume. Final primer and probe concentrations (purchased at Eurofins MWG Operon, Ebersberg, Germany) in ddPCR mixes were identical to the qPCR conditions used in this study, and to those used in the previously described chamber digital PCR (cdPCR) conditions [11] (see Appendix S1 and Table S2).

ddPCR workflow and data analysis were performed as described (see Appendix S1) [15].

### Determination of ddPCR key performance parameters

#### Comparison of singleplex and duplex reactions

The ddPCR duplex assay was evaluated using three 8-well cartridges containing the singleplex *hmg*, the singleplex MON810 and the duplex ddPCR assay respectively. In each cartridge, one well was filled with a non template control (NTC) ddPCR mix, while the seven other wells contained ddPCR mixes with DNA extracted from the ERM-BF413ek CRM (average of 46,571 *hmg* copies and 324 MON810 copies). Droplets were generated for each individual cartridge, and those droplets containing the PCR mixes of the three cartridges were transferred onto a single PCR plate for amplification followed by droplet count.

#### Dynamic range, repeatability, limits of detection and quantification

A dilution series was prepared with MON810 maize DNA extracted from the ERM-BF413gk CRM. DNA quantification in the initial MON810 maize DNA solution was estimated by qPCR as described [23]. The dilution series consisted of 14 solutions containing from approximately 118000 to 0.02 copies of *hmg*, and from 4300 to 0.0008 copies of MON810 per 20 µL, respectively). Five replicates of the dilution series and of a non template control (NTC) were measured by ddPCR. For qPCR, measurements were made in duplicate. Linearity over the dynamic range was determined by the coefficient of correlation R^2^, calculated on the average of the target copy numbers measured in the replicated dilution series for both qPCR and ddPCR. Repeatability over the dynamic range was determined by the coefficient of variation (cv) of the measured target copy number or of the MON810 content between the replicates of the dilution series. The absolute limit of quantification (aLOQ) and absolute limit of detection (aLOD) for qPCR and ddPCR were determined on these experimental results.

An additional set of experiments was performed to establish repeatability between different emulsification runs. Intra- and inter-cartridge (ddPCR 8 well chips) repeatability was determined on five independent series consisting of seven replicates of the ERM-BF413ek (approximately 100 ng and an average of 46571 *hmg* copies and 324 copies of MON810 per 20 µl reaction) and one NTC. Four series were prepared by two operators and droplet amplification reactions were performed simultaneously on the same 96-well plate. The fifth series was prepared by one operator on a second day and ddPCR reactions were performed on another 96-well plate. A total of 35 replicate positive ddPCR results were then analyzed.

#### Specificity

The DNA extracts from samples G053/12 (approximately 6,200 copies of *hmg*) and G031/12 (approximately 100 ng DNA) were tested with the duplex MON810/*hmg* ddPCR assay. A total of eight replicate reactions per sample were performed (see Appendix S1 for more details).

#### Applicability

Samples representative of four different maize-containing matrices from routine GMO testing were used to test the applicability: seed-powder flour, corn flakes, wheat seed-powder flour with maize contamination, and feed containing maize (see Appendix S1 and Table S1).

## Results and Discussion

Given the limitations of qPCR for the quantification of GMO in food and feed samples, especially at low target levels and in some complex matrices, the use of ddPCR in routine GMO quantification was evaluated, following the generally accepted minimum performance requirements for analytical methods [24], [25].

In order to avoid biases as much as possible when comparing qPCR and ddPCR quantification, we transferred the inter-laboratory validated qPCR *hmg*- and MON810-specific assays to ddPCR technology with the minimum of adaptation. Therefore, apart from the mastermix and settings that are specific to the QX100 droplet system, primers and probe nucleotide sequences and concentrations, DNA concentration, and PCR thermo-profile were kept identical to those in the qPCR assays.

### ddPCR can readily be set as a duplex application

Because GMO content is calculated based on the ratio of transgene/endogene quantities, it would be more practical to perform endogene–transgene duplex reactions to reduce costs. For this reason, evaluation of duplex qPCR and ddPCR assays was performed and compared to the performance of singleplex assays. The primers and probes of both the *hmg* and MON810 systems were mixed in the qPCR or ddPCR volumes to final concentrations equal to those in the original singleplex assays.

Evaluation of qPCR duplex systems has shown that the *hmg* system performed identically in duplex and singleplex qPCR reactions, while MON810 amplification was significantly affected in the duplex reactions, showing signal values approximately 5.5 Cq higher than in singleplex reactions (data not shown). An attempt to optimize the duplex qPCR assay was made by varying primers and probe concentration of both systems. However, the *hmg* and MON810 were affected differently under all the tested conditions, resulting in under-estimations of the MON810 content and/or loss of sensitivity (data not shown). These results are not surprising: the difficulty of multiplexing qPCR assays is well documented, including its application to GMO quantification [26]–[28]. One of the limitations is the need to choose target sequences with similar, short lengths. Another difficulty linked to GMO detection is that the event-specific targets needed for reliable and specific GMO quantification are usually the junction regions between the transgene and the host plant genome, leaving a very narrow window for design, and decreasing further the flexibility for a multiplex design. Also, the need is usually to quantify low concentrations of transgene (down to 0.1%) in a background of high endogene quantities. This asymmetry in concentration renders establishment of a qPCR duplex assay targeting both the MON810 transgene and the *hmg* endogene even more difficult. In the following experiments, results of only the singleplex qPCR assays were used for comparison with ddPCR assay results.

For ddPCR, no significant variation of the measured target copy number was observed between the singleplex and duplex ddPCR assays for both *hmg* (bias = −1.8%) and MON810 systems (bias = 3.7%). Similarly, no significant variation of the measured MON810 content was observed between the singleplex and the duplex ddPCR assays (bias = 5.8%) (Table S3). Additionally, the repeatability of the duplex assay in measurements of the MON810 content appears slightly better that of the singleplex assays. From these results, it was concluded that the duplex ddPCR assay performs as well as the singleplex ddPCR assays without any additional optimization. This was further confirmed by the successful establishment of several additional duplex ddPCR assays from singleplex qPCR assays, targeting GM events or screening elements, without optimization (data not shown).

### Enzymatic restriction of genomic DNA

It was recently suggested that it is preferable to expose gDNA to endonuclease restriction in order to improve amplification efficiency and to increase the accuracy of GMO target copy number measurement with cdPCR [19]. gDNA endonuclease restriction for ddPCR testing was also evaluated and found unnecessary (Table S4).

Digestion of DNA may be necessary for digital PCR analysis in the case of a possible linkage between targets, such as multiple copies of target physically linked on the same chromosome, or if different targets present on a same plasmid need to be quantified. In the case of GMO quantification, the target of the event-specific assay and the target chosen for endogene quantification are present as unique copies in the host plant genome and are not linked. In the case of MON810 maize, results of the two dimensional analyses of the droplet signals in digested and undigested DNA samples (Figure S1 in Supporting Information) suggests the absence of linkage between MON810 positive droplets and *hmg* positive droplets, thus confirming the independence of the two targets. Further, ddPCR performance was evaluated using non-digested gDNA.

### Dynamic Range, precision and limits of quantification

A recent study has estimated that the theoretical ddPCR dynamic range is 10^5^ target copies, and it has been established experimentally that the dynamic range covers more than 4 orders of magnitude [15].

The ddPCR duplex assay response was investigated over target concentrations ranging from approximately 0.02 to 118,000 *hmg* copies and from approximately 0.0008 to 4300 MON810 copies per 20 µL of ddPCR reaction. Due to pipetting errors, that were noted after loading the 8-well cartridges with ddPCR mixes, data from one reaction (1.4% of the total dataset) were excluded from the analysis. The average number of droplets read for each ddPCR reaction included in the data analysis was 13,606 with a standard deviation of 931 droplets (coefficient of variation cv = 6.8%).

**Figure 1:**
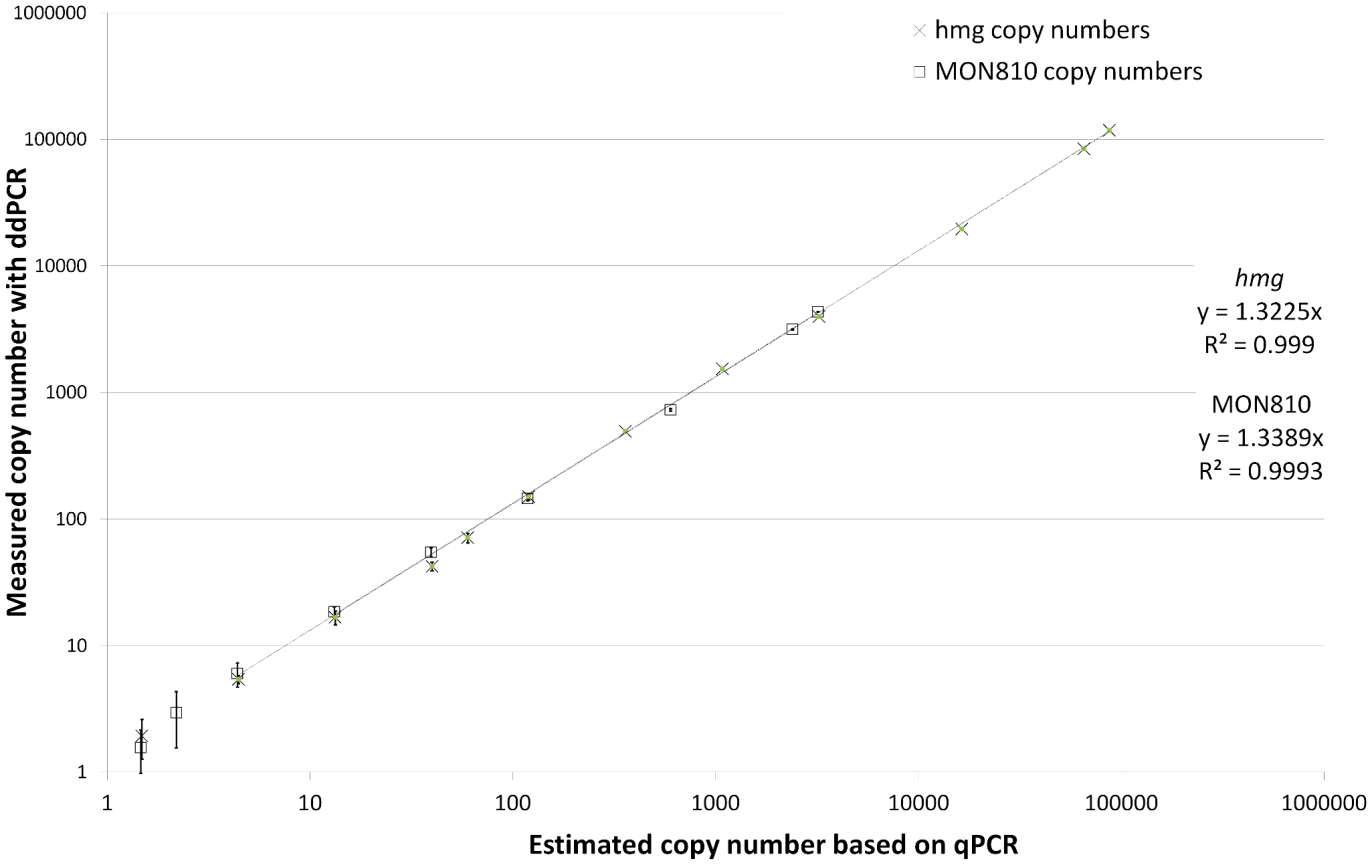
Dynamic range of the ddPCR duplex assay. Five replicates for each data point. Error bars represent the standard deviation between the five replicates at each target concentration.

The ddPCR response was linear over concentrations ranging from an average of 5 to 118,000 *hmg* copies (0.02% to 99.5% positive droplets) with a coefficient of correlation (R^2^) of 0.9990. Similarly, the ddPCR response for MON810 was, on average, linear from 6 to 4,340 MON810 copies (R^2^ = 0.9993; 0.03% to 17.9% positive droplets in average) (Figure 1). This performance was similar to those of the two singleplex qPCR assays, which was linear over the same dynamic ranges (R^2^ *hmg* = 0.9939 and R^2^ MON810 = 0.9958) (data not shown). The ddPCR linear response for the MON810/*hmg* duplex assays covered a broader range than the same assays tested in cdPCR which was limited to 2–3 orders of magnitude [18], [19]. This wider range of concentrations can be attributed to the large number of partitions available for reactions in ddPCR (13,606 droplets on average in this work) compared to the number (765) available for cdPCR test. It has already been asserted that qPCR offers a much broader dynamic range than digital PCR [16]. The dynamic range observed for ddPCR covers the whole range of target concentrations usually needed by a laboratory for routine GMO testing (0.1% to 100% transgene/endogene ratio cp/cp).

For individual targets and for GMO content, the coefficient of correlation R^2^ obtained with ddPCR met the requirements (R^2^>0.98) set by the European Union Reference Laboratory for GM Food and Feed [25] for acceptance of a quantitative PCR-based detection method for GMO.

All samples used for determining the dynamic range came from serial dilution of a single stock MON810 maize DNA sample. At a higher concentration (118,000 *hmg* copies per 20 µl ddPCR mix), each droplet contained, on average, 5.9 *hmg* molecules, which is the upper recommended concentration for use of the droplet system (Bio-Rad, personal communication). This finding supports the fact that the duplex MON810/*hmg* ddPCR assay can be used over a wide range of target concentrations to determine the MON810 content in a given sample, and that values around 115,000 copies constitute the upper range of quantification with ddPCR.

The absolute limit of quantification (aLOQ) is the lowest target copy number in a sample that can be reliably quantified with an acceptable level of precision and accuracy [25]. The aLOQ of the *hmg* or MON810 ddPCR systems was estimated as the lowest copy number within the dynamic range with a coefficient of variation (cv) of the measured copy number≤25% [25]. Based on this criterion, aLOQ was estimated to be around 5 copies for the *hmg* system, and 18 copies for the MON810 system and for the duplex ddPCR assay (Table S5). As a comparison, it is usually agreed that aLOQ of qPCR assays range from 30 to 100 target copies per reaction [12], [29]. The aLOQ of the qPCR MON810 specific method used in this study was initially estimated at 10 copies of the target MON810 sequence [22], and at a higher range of 31–63 copies in another recent study [18]. In our laboratory, the aLOQ estimated for the qPCR assay on the same DNA is 18 copies. The aLOQ of the duplex ddPCR assay was therefore equal to or better than that estimated for the qPCR performance. Similarly, the aLOQ of the duplex ddPCR assay was also in a range similar to the aLOQ measured in cdPCR (15–56 transgene copies), assessed only on the MON810 assay [18].

### Sensitivity

The absolute limit of detection (aLOD) is the lowest target copy number in a sample that can be reliably detected, but not necessarily quantified [25]. For this study, aLOD was calculated based on experimental data obtained to determine the dynamic range. aLOD was determined as the lowest concentration level for which all five ddPCR replicates resulted in at least two positive droplets per reaction. aLOD was estimated to be five copies for the *hmg* system, and six copies for the MON810 system, which is suitable for routine GMO testing. The absolute sensitivity is, according to our own assessment (data not shown), lower than the one observed for the MON810 singleplex qPCR assay at around 6–18 copies, and comparable to the one observed in cdPCR [18].

### Repeatability

**Figure 2:**
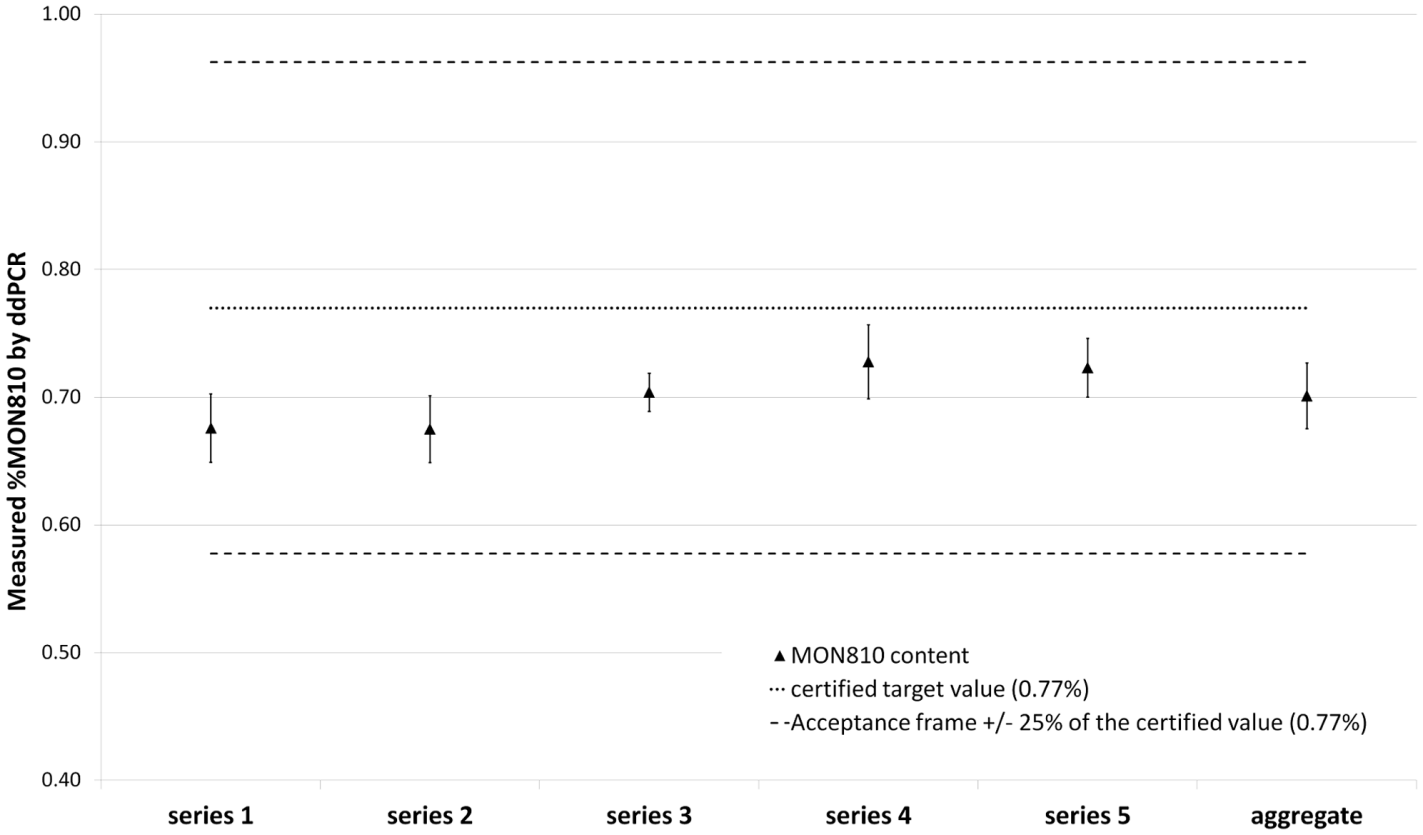
Repeatability results of the ddPCR duplex assay. MON810 content measured by ddPCR in five series of seven replicates. The aggregate represents the sum of the five series. The target certified MON810 content (0.77%) is indicated by a dotted line. Acceptance criterion for repeatability is ±25% of the target content (from 0.58% to 0.96%) represented by the dashed lines. Error bars represent the standard deviation between the replicates for each series or in the aggregate.

Intra- and inter-cartridge repeatability of the ddPCR was assessed by two different operators and over two different days for both *hmg* and MON810 target copy number determinations and for MON810 content determination. Less than 10% variability was observed within each of the five cartridges for the determination of *hmg* copies, MON810 copies, and MON810 content. Similarly, comparison of the values between the five cartridges showed low variability (cv<10%) for all the three measured parameters (Figure 2 and Table S6).

The overall repeatability could also be estimated by analyzing the results of the experiment performed for aLOQ and dynamic range determination. All along the dynamic range, the cv of the determined *hmg* copies, MON 810 copies, and MON810 content remained below the threshold for acceptance of quantitative methods (cv<25%) (Table S5).

**Figure 3:**
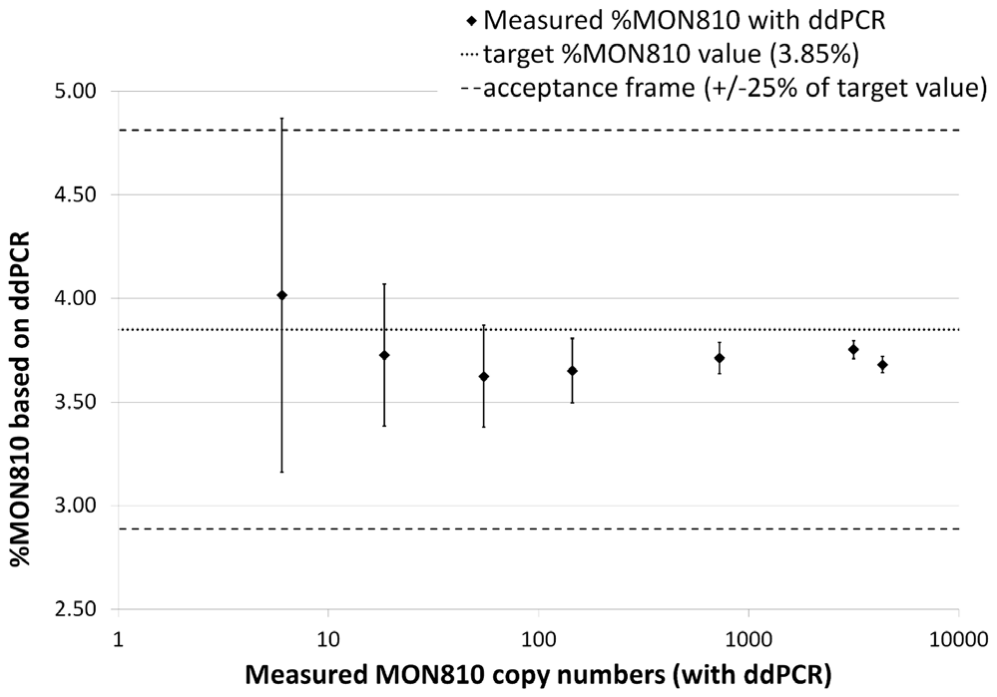
Precision of the duplex ddPCR assay as a function of the target concentration. MON810 content measured by ddPCR in five series of seven target concentrations. The target MON810 content (3.85%) is indicated by a dotted line. Acceptance criterion for precision is ±25% of the target content (from 2.89% to 4.81%) represented by the dashed lines. Error bars represent the standard deviation of the measured MON810% by ddPCR at each target concentration (five replicates per target concentration).

It has already been observed with both ddPCR [15] and cdPCR [19] that the relative uncertainty in target copy number varies across the dynamic range, with higher uncertainty, and consequently higher measurement variability, as the target copy number decreases. In this study, similar variability of the measured target copy numbers (Table S5 and Figure 1) and of the MON810 content was observed at lower target concentrations (Table S5 and Figure 3).

In all experiments and for all three parameters (hmg and MON 810 copy number, MON810 content), the coefficient of variability measured at each point of the dynamic range was far below the 25% threshold set in international guidance documents for validation of GMO testing methods [24], [25]. These experiments demonstrate that, using ddPCR, one can obtain repeatable and comparable quantitative estimates of GMO target number or content.

### Trueness

Trueness is defined as the closeness of agreement between the average value obtained from a series of test results and an accepted reference value [25]. Acceptance criterion for trueness is that the measured value has to be within ±25% of the accepted reference value over the whole dynamic range [24], [25].

To assess trueness, data generated by the experiments for dynamic range determination and for intra- and inter-cartridge repeatability performance were used. In the absence of a DNA reference material certified for absolute copy number concentration, trueness could only be assessed for the MON810 content.

In the experiment performed to determine the intra- and inter-cartridge repeatability (on CRM ERM-BF413ek), the average value of the pooled ddPCR data showed good agreement with the certified value (Table 1) and, in every case, the MON810 content measured by ddPCR (within the dynamic range) was within ±25% of the certified value (Figure 2). By comparison, the MON810 content measured by qPCR was close to the limit of acceptance (Table 1).

**Table 1:** Application of the ddPCR duplex assay on different sample matrices.

Sample	Target value	Average MON810% (ddPCR)	Bias MON810% (ddPCR)	Average MON810% (qPCR)	Bias MON810% (qPCR)
ERM-BF413d	0.57%^a^	0.62%	8.0%	0.46%	−19.3%
ERM-BF413f	2.85%^a^	2.92%	2.5%	2.29%	−19.6%
ERM-BF413ek	0.77%^a^	0.70%	−9.0%	0.58%	−24.7%
ERM-BF413gk	3.85%^a^	3.68%	−4.1%	3.66%	−4.9%
G0009/04	0.29%^c^	0.26%	−11.7%	0.29%	n.a.
G0180/07	0.04%^c^	0.04%	2.9%	0.04%	n.a.
G211/10	0.45%^b^	0.46%	−1.8%	0.50%	11.1%
G212/10	2.10%^b^	2.32%	10.4%	2.30%	9.5%
G231/11	2.64%^c^	2.31%	−12.4%	2.64%	n.a.
G0147/08	29.6%^c^	21.7%	−26.7%	29.6%	n.a.
G254/11	3.82%^c^	3.47%	−9.2%	3.82%	n.a.

Target value: MON810 content expressed as a percentage in cp/cp ratio.

a Target value certified by the CRM provider or evaluated from the certified value of another CRM in the same series. ^b^Target value in cp/cp ratio attributed in the ILC-CRL-GMFF proficiency program. ^c^Target value estimated by qPCR, using a CRM certified in cp/cp ratio (same as “Average MON810% (qPCR)”).

Average MON810% (ddPCR): Average of the MON810 content measured by ddPCR.

Bias MON810% (ddPCR): Bias of the average MON810 content measured by ddPCR with the target value.

Average MON810% (qPCR): Average of the MON810 content measured by qPCR.

Bias MON810% (qPCR): Bias of the MON810 content measured by qPCR with the target certified value.

n.a.: not applicable (value determined by qPCR is the target value).

The average value of the pooled ddPCR duplex assay data at each dilution level of the dynamic range showed good agreement with the reference value of 3.85% cp/cp (ERM-BF413gk) (Table 1). The MON810 content measured by qPCR was similar to that of the ddPCR value, the latter being slightly closer to the target value (Table 1). Throughout the dynamic range, each individual ddPCR measurement of the MON810 content fell within ±25% of the certified value (Figure 3). It is noteworthy that the deviation between the MON810 content measured by ddPCR and the reference value tended to increase with lower target copy number. Nevertheless, the MON810/hmg ddPCR duplex assay met trueness acceptance criteria throughout the whole dynamic range. Trueness was also evaluated on two additional CRMs and on samples from the ILC-EURL-GMFF proficiency program: ddPCR results showed better agreement with the target values than qPCR and were in accordance with the trueness acceptance criterion (Table 1).

### Specificity

The ddPCR amplicons used for this study are the same as for the qPCR singleplex assays that were subjected to inter-laboratory validation and for which specificity was thoroughly checked at this stage. Moreover, the key assay parameters (primer and probe concentration, thermal profiles) were not modified. Therefore, the specificity of the ddPCR amplicons was not thoroughly verified as it is inferred that specificity should be the same as for the qPCR singleplex assays. A limited specificity study was however conducted on two samples containing either wild-type maize, or DNA from milk sample without maize. In all cases, a false-positive rate far below 5%, which is the generally used acceptance criterion, was observed (Table S7). In addition, for all experiments performed in this study, NTC resulted in no signal (no positive droplet).

### Applicability

Another important factor when introducing new methods or technologies for testing GMOs in food and feed is their applicability. More specifically, their ability to perform well with different sample matrices and within a range of concentrations relevant for GMO testing has to be demonstrated [24].

MON810 contents measured by the ddPCR duplex MON810/*hmg* assay in maize seed-powder flour samples and corn flakes samples are in good accordance with the values measured with the qPCR singleplex MON810 and *hmg* assays (Table 1).

During qPCR tests, we detected the presence of inhibition in the stock DNA solution of two samples (wheat seed-powder flour with maize contamination and maize feed), as indicated by the large differences in MON810 content (cv>25%) calculated from different sample dilutions. Consequently, diluted DNA samples were used to determine the MON810 content with qPCR. In the wheat sample, the generally used NucleoSpin Food kit (NSF) extraction protocol resulted in strong inhibition of the qPCR amplifications, and new DNA extraction following the CTAB protocol was needed. Both stock and diluted DNA solutions from the CTAB extract could be used for MON810 content determination with qPCR. The ddPCR measurements of MON810 content were in agreement with the accepted values obtained with qPCR but with a bias slightly above the acceptance limit for the wheat seed-powder flour sample (Tables 1 and S6). It has to be noted that there is substantial empirical knowledge about the efficacy of DNA extraction methods related to sample matrix. GMO laboratories use adapted DNA extraction methods that in some cases may reduce inhibition during the qPCR analysis. Furthermore, inhibition is already assessed during the screening and identification phases of GMO testing, so there is no risk that substances in a DNA extract would totally inhibit the qPCR or ddPCR amplification reaction, resulting in false negatives.

Interestingly, for the DNA extracts obtained with NSF, very low differences were observed between the MON810 content determined by ddPCR in the stock (presenting inhibition with qPCR) and the diluted DNA solutions for both matrices (Table S8 in Appendix S2). This result suggests that the ddPCR duplex assay is more tolerant to inhibitors found in some complex food and feed matrices than the qPCR assays, as suggested earlier [18].

In summary, the ddPCR MON810/*hmg* duplex assay can be applied for routine quantification of the MON810 maize, as demonstrated on a large range of transgene content (experimentally from 0.04% to 29.6%) usually found in samples. Moreover, its use for several types of food, feed and seed matrices commonly found in routine samples has been verified.

### Practicability

Before introducing a new technology in a laboratory, one has to verify its practicability for daily use [24].

To do so, calculations were made based on the simultaneous quantification of four samples-the average number run in parallel in middle-size GMO laboratories. The quantitative analysis of four samples using *hmg* and MON810 singleplex qPCR assays requires a total of 96 reactions (see set up A, Table S9 in Appendix S2). This number of reactions is high, due mainly to the use of inter-laboratory validated singleplex methods, the need for monitor inhibition that requires additional dilutions, and the need for a standard curve. With ddPCR, the standard curve is not needed and a duplex MON810/*hmg* assay with a wide dynamic range is used. Since inhibition is assessed during the screening and identification phases of GMO testing and given the demonstrated tolerance of the assay for amplification inhibition, it is not necessary to control the inhibition at the ddPCR quantification stage. Thus, a simple ddPCR testing set-up is proposed (set up B, Table S9 in Appendix S2) that included two replicate reactions for each test portion of the sample, in accordance with the ISO 21570:2005 standard [30]. Including NTC and quantification control reactions in the set-up, a total of 20 reactions would be necessary to reliably quantify MON810 in four samples.

Based on the experience acquired during this study and assuming the samples and mixes are already prepared, the simultaneous analysis of these four samples with ddPCR would require approximately 190 minutes. For comparison, qPCR would take 160 minutes to generate results (see Table S10 in Appendix S2). In terms of hands-on-time, ddPCR would require approximately 15 min less than qPCR. The main difference between the two approaches can be attributed to the time needed by the droplet reader to analyze individual droplets.

Considering the cost of reagents, consumables and labor at NIB and the above proposed set-ups for GMO testing, the quantification of a given transgene in four samples with ddPCR would cost approximately US$20.9 per sample and US$22.3 using qPCR. If more samples must be handled simultaneously, ddPCR shows even better throughput and smaller cost than qPCR (see Tables S11 and S12 in Appendix S2). The use of cdPCR, taking into account prices per chip or plate (from US$150 to US$400 each) [16] and using the proposed set-up for ddPCR (four runs, i.e. chips or plate per sample), would lead to a cost per sample much greater than that required for current routine detection of GMOs in most laboratories.

Quantification of routine samples using ddPCR is therefore practical and has the potential to provide better throughput and cost-effectiveness than qPCR for GMO laboratories. A MIQE checklist is made available in Appendix S3.

## Conclusion

The intention of this study was to demonstrate the usability of ddPCR in real-life routine diagnostics, rather than to re-investigate the recently reported metrological characteristics of this technology [15]. The applicability of ddPCR was investigated for the quantification of GMO in food, feed and seed samples. The ddPCR MON810/*hmg* duplex assay presented here and implemented without optimization from the inter-laboratory validated singleplex qPCR assays, achieves a wide dynamic range close to five orders of magnitude with an upper limit of quantification of about 118,000 target copies. It also shows very good sensitivity, suitable for GMO testing. The excellent performance of the tested parameters enables the quantification of samples from different matrices, using DNA extracted with common methods without up-front DNA quantity estimation. The limits of quantification, trueness and repeatability of the duplex assay comply with international recommendations [24], [25] and are comparable or superior to those of the inter-laboratory validated qPCR singleplex assays (Table 2).

**Table 2:** Summary table of qPCR, ddPCR and cdPCR performance for MON810 detection and quantification.

Parameter tested	qPCR	ddPCR	cdPCR
“plexing” of the assay	Singleplex	Duplex	Duplex
Need for endonuclease treatment	No	No	Yes
Dynamic range	5 logs	5 logs	2–3 logs [18], [19]
Absolute limit of quantification	18 copies (MON810, this study), 30–60 copies [18]	5 copies (*hmg*), 18 copies (MON810)	15–56 copies (MON810) [18]
Absolute limit of detection	1–10 copies (this study and [18])	5 copies (*hmg*), 6 copies (MON810)	1–10 copies (MON810) [11], [18], [19]
Repeatability through the dynamic range	Cv<35% (%Mon810) [22]	Cv<25% (*hmg* cp nb), Cv<20% (Mon810 cp nb), Cv<19% (%Mon810)	Cv<11% (*hmg* cp nb), Cv<22% (Mon810 cp nb), Cv<23% (%Mon810) [11]
Trueness	From −16.7% to 2.3% [22]. From −24.7% to 11.1% (this study)*	From −9.0% to 10.4% (this study)*	From −21.20 to 41.4% [18]
Applicability			
- *Matrices tested in this study*	Food, feed and seeds	Food, feed and seeds	Seeds, plasmid
- *GMO content range tested in this study*	0.04% to 26.4%	0.04% to 26.4%	0.07% to 57.1% [11]
- *Sensitivity to inhibitors*	Sensitivity to inhibition (use dilutions, alternative DNA extraction)	Less (not?) sensitive to inhibition	N.A.
Practicability			
- *Number samples/96-well plate*	4 samples	23 samples	N.A.
- *Time for results/96-well plate*	3 hours	6 hours	N.A.
- *Price/sample if 96-well plate*	US$23.3	US$16.1	N.A.

qPCR: data produced in this study, or obtained from the literature, when indicated [18], [22]

ddPCR: data produced in this study.

cdPCR: data produced on a BioMark System (Fluidigm, South San Francisco) using the 12.765 digital arrays (Fluidigm) and obtained from the literature [11], [18], [19].

Repeatability through the dynamic range: assessed through the coefficient of variation (Cv) of the target copy numbers or the MON810 content between repeats.

Trueness: assessed through the calculation of the bias between the MON810 content measured and the target MON810 content. * For our study, trueness is indicated only when qPCR and ddPCR results could be compared to a third, independent value (obtained from the CRM provider or proficiency test organizer).

Time for results/96 well plate: Total time needed from DNA pipetting to the analysis of the results; reaction mixes are already prepared.

Price/sample if 96-well plate: Price based on material and reagent costs available at NIB, including labor cost.

N.A.: not evaluated.

Applicability of the technology has been verified on representative matrices found in routine samples, and on the range of GMO content usually found in routine samples and relevant to different international labeling requests. Unlike qPCR, quantification by ddPCR has been found to be insensitive to the amplification inhibition present in some DNA extracts. It is also very precise at very low levels of target content. The use of the ddPCR duplex assay in routine GMO analysis was shown to be practical, following the new test set-up proposed in this study.

It was recently discussed as to whether price is a limiting factor to the adoption of dPCR in the laboratory [16]. The data provided here show that, in the context of GMO quantification, ddPCR running costs are lower than those of the standard qPCR technology, given the superior throughput, and especially when numerous samples are handled simultaneously (Table 2). Increasing the multiplexing will certainly give further, additional advantage to ddPCR in terms of cost and throughput, and could allow its use already at the screening and/or identification steps. The establishment of duplex reactions is straightforward and does not need optimization, which is also encouraging. This characteristic has the advantages of reducing the cost of analysis, and of decreasing the uncertainty linked to droplet volume variation and dilution pipetting errors [19].

To be employed in routine testing, methods based on ddPCR shall be properly validated through ring-trials and verified during their introduction in laboratories to demonstrate their fitness for the purpose. However, the ddPCR performance demonstrated in this study on real routine samples should lead to greater confidence and easier adoption of digital PCR technology, to generating more precise data on everyday tests, and at overall better cost per sample. The demonstrated application of ddPCR for routine quantification of GMO content in food and feed samples should act as an inducement to introduce this technology in other areas where precise analytical testing is required in food and feed samples.

## Supporting Information

Figure S1 **VIC** ***vs*** **. FAM channel clustering plot of droplets for non-digested and** ***Taq*** **I digested MON810 DNA.** Upper frame: Non digested DNA. Lower frame: *Taq*I digested DNA. Upper left quadrant: FAM (*hmg*) positive-VIC (MON810) negative droplet cluster. Upper right quadrant: FAM (*hmg*) positive-VIC (MON810) positive droplet cluster. Lower left quadrant: FAM (*hmg*) negative-VIC (MON810) negative droplet cluster. Lower right quadrant: FAM (*hmg*) negative-VIC (MON810) positive droplet cluster.
(TIF)

Table S1 **Test material used in this study.**
(DOC)

Table S2 **Primers and probes used in this study.**
(DOC)

Table S3 **Comparison of quantification using singleplex and duplex ddPCR assays.**
(DOC)

Table S4 **Comparison of duplex ddPCR quantification on digested and non-digested genomic DNA.**
(DOC)

Table S5 **Results from the dilution series used for the dynamic range, the aLOD and aLOQ determination, and the overall repeatability.**
(XLS)

Table S6 **Inter- and intra-cartridge repeatability.**
(XLS)

Table S7 **False-positive rates observed with ddPCR.**
(DOC)

Appendix S1 **Supporting material and methods.**
(PDF)

Appendix S2 **Table S8. Inhibition effect on MON810 quantification in qPCR and ddPCR.** Table S9. Set-up and number of reactions needed for simultaneous quantification of four samples with qPCR and ddPCR. Table S10. Time needed for quantification with qPCR and ddPCR. Table S11. Set-up and number of reactions needed for simultaneous quantification of 23 samples with qPCR and ddPCR. Table S12. Set-up and number of reactions needed for quantification with qPCR and ddPCR (full 96 well-plate).
(XLS)

Appendix S3 **MIQE checklist.**
(PDF)
