# Supplementary figures and images for: Quantitative Analysis of Food and Feed Samples with Droplet Digital PCR
Source: PLoS One. 2013 May 2;8(5):e62583. doi: 10.1371/journal.pone.0062583 (PMC3642186; doi:10.1371/journal.pone.0062583)

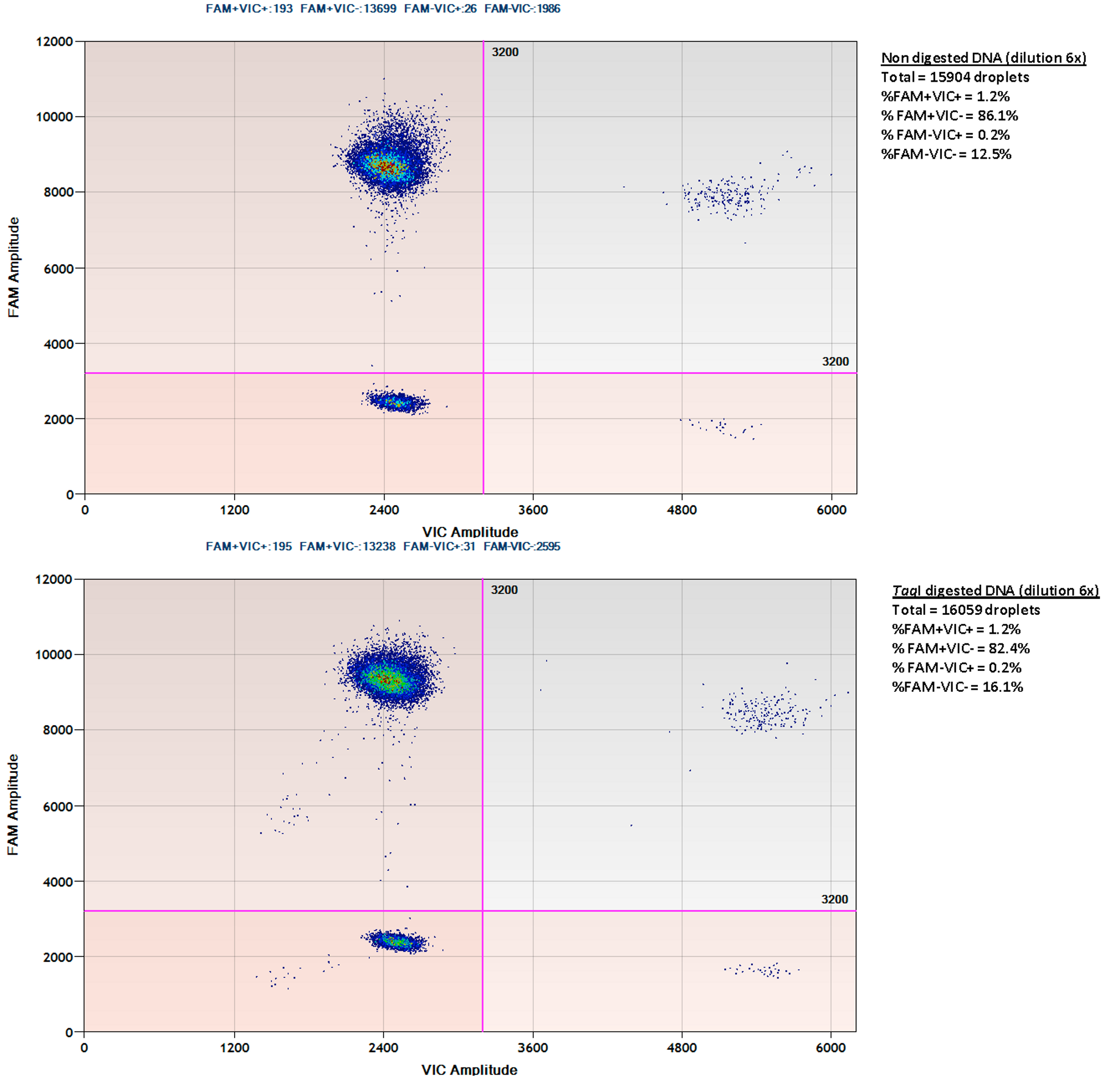

Supplement: Figure S1 — VIC vs . FAM channel clustering plot of droplets for non-digested and Taq I digested MON810 DNA. Upper frame: Non digested DNA. Lower frame: TaqI digested DNA. Upper left quadrant: FAM (hmg) positive-VIC (MON810) negative droplet cluster. Upper right quadrant: FAM (hmg) positive-VIC (MON810) positive droplet cluster. Lower left quadrant: FAM (hmg) negative-VIC (MON810) negative droplet cluster. Lower right quadrant: FAM (hmg) negative-VIC (MON810) positive droplet cluster. (TIF) [file pone.0062583.s001.tif]
