# Supplementary material for: Quantitative Analysis of Food and Feed Samples with Droplet Digital PCR
Source: PLoS One. 2013 May 2;8(5):e62583. doi: 10.1371/journal.pone.0062583 (PMC3642186; doi:10.1371/journal.pone.0062583)
Supplement: Table S1 — Test material used in this study. (DOC) [file pone.0062583.s002.doc]

Table S-: Test material used in this study

| **Sample** | **Source** | **Matrix** | **Mon810 content (m/m)** | **Mon810 content (cp/cp)** |
| --- | --- | --- | --- | --- |
| ERM-BF413d | CRM | Seed-powder flour | 1% ±0.3% ^a^ | 0.57% ±0.17% ^a^ |
| ERM-BF413f | CRM | Seed-powder flour | 5% ±0.2% ^a^ | 2.85% ^d^ |
| ERM-BF413ek | CRM | Seed-powder flour | 1.98% ±0.15% ^a^ | 0.77% ±0.08% ^a^ |
| ERM-BF413gk | CRM | Seed-powder flour | 9.9% ±0.5% ^a^ | 3.85% ^d^ |
| G0009/04 | USDA/GIPSA PP | Seed-powder flour | 0.30% ^b^ | 0.29% ±0.13% ^d^ |
| G0180/07 | USDA/GIPSA PP | Seed-powder flour | < 0.1% ^b^ | 0.04% ±0.02% ^d^ |
| G211/10 | ILC-EURL-GMFF PP | Seed-powder flour | 0.81% ±0.07% ^b^ | 0.45% ±0.098% ^b^ |
| G212/10 | ILC-EURL-GMFF PP | Seed-powder flour | 3.83% ±0.17%^b^ | 2.10% ±0.35%^b^ |
| G147/08 | Gemma PP | Seed-powder flour | 51.9% ±15.6% ^c^ | 29.6% ±8.9%^e^ |
| G231/11 | Routine | Corn flakes | 4.63% ±1.4% ^c^ | 2.64% ±0.8%^e^ |
| G254/11 | Routine | Feed | 6.71% ±2.0% ^c^ | 3.82% ±1.1%^e^ |
| G031/12 | Routine | Milk sample | n.d. | n.d. |
| G053/12 | Routine | Feed | n.d. | n.d. |

Source: source of the sample. CRM: Certified Reference Material. PP: Proficiency program.

MON810 content (m/m): MON810 content expressed as a percentage in mass to mass ratio.

^a^ Value certified by the CRM provider. ^b^ Value attributed in the proficiency program. ^c^ Value estimated by qPCR, using a CRM certified in m/m ratio.

MON810 content (cp/cp): MON810 content expressed as a percentage in copy to copy ratio.

^d^ Value evaluated using another CRM in the same series, certified in cp/cp ratio. ^e^ Value estimated by qPCR, using a CRM certified in cp/cp ratio.

n.d.: not detected.
