# Supplementary material for: Quantitative Analysis of Food and Feed Samples with Droplet Digital PCR
Source: PLoS One. 2013 May 2;8(5):e62583. doi: 10.1371/journal.pone.0062583 (PMC3642186; doi:10.1371/journal.pone.0062583)
Supplement: Table S2 — Primers and probes used in this study. (DOC) [file pone.0062583.s003.doc]

**Table S-**: Primers and probes used in this study

| **Target DNA** | **Primer/probe name** | **Primer/probe sequence (5’ – 3’)** | **Final concentration (nM)** |
| --- | --- | --- | --- |
| *hmg* | Fw-hmg | TTGGACTAGAAATCTCGTGCTGA | 300 |
|  | R-hmg | GCTACATAGGGAGCCTTGTCCT | 300 |
|  | P-hmg | HEX- CAATCCACACAAACGCACGCGTA-BHQ-1 | 180 |
| MON810 | Fw-MON810 | TCGAAGGACGAAGGACTCTAACGT | 300 |
|  | R-MON810 | GCCACCTTCCTTTTCCACTATCTT | 300 |
|  | P-MON810 | 6-FAM- AACATCCTTTGCCATTGCCCAGC - BHQ-1 | 180 |

6-FAM: 6-carboxyfluorescein, BHQ1: Black Hole Quencher 1, HEX: hexachloro-6-carboxyfluorescein
