# Supplementary material for: Quantitative Analysis of Food and Feed Samples with Droplet Digital PCR
Source: PLoS One. 2013 May 2;8(5):e62583. doi: 10.1371/journal.pone.0062583 (PMC3642186; doi:10.1371/journal.pone.0062583)
Supplement: Table S3 — Comparison of quantification using singleplex and duplex ddPCR assays. (DOC) [file pone.0062583.s004.doc]

Table S-: Comparison of quantification using singleplex and duplex ddPCR assays

| **Analyte** | **Average**  **(duplex)** | **Cv**  **(duplex)** | **Average**  **(singleplex)** | | **Cv**  **singleplex)** | | **Bias**  **duplex/singleplex** | |
| --- | --- | --- | --- | --- | --- | --- | --- | --- |
| *hmg* copies | 47,086 | 1.3% | 47,971 | 1.6% | | -1.8% | |  |
| MON810 copies | 340.5 | 7.2% | 328.0 | 9.4% | | 3.7% | |  |
| MON810% | 0.72% | 6.4% | 0.68% | 6.9% | | 5.8% | |  |

Average: average from seven measurements, expressed in copy numbers (*hmg* and MON810 copies) or %MON810.

Cv: coefficient of variability between the seven measurements. Expressed as a percentage of the average value.

Bias duplex/singleplex: Bias of the average values obtained from the duplex reactions in comparison with the average values obtained by singleplex ddPCR reactions. Expressed as a percentage of the target value.
