# Supplementary material for: Quantitative Analysis of Food and Feed Samples with Droplet Digital PCR
Source: PLoS One. 2013 May 2;8(5):e62583. doi: 10.1371/journal.pone.0062583 (PMC3642186; doi:10.1371/journal.pone.0062583)
Supplement: Table S4 — Comparison of duplex ddPCR quantification on digested and non-digested genomic DNA. (DOC) [file pone.0062583.s005.doc]

Table S-: Comparison of duplex ddPCR quantification on digested and non-digested genomic DNA

| **Analyte** | **Average (native)** | **Cv copy number (native)** | **Average (digested)** | **Cv copy number (digested)** | **Bias non-digested/digested (average)** |
| --- | --- | --- | --- | --- | --- |
| *hmg* copies | 264,537 | 3.3% | 204,924 | 16.8% | 29.1% |
| MON810 copies | 1,868 | 5.9% | 1,460 | 21.7% | 27.9% |
| MON810% | 0.71% | 6.7% | 0.71% | 7.4% | -0.28% |

Average: average from four measurements, expressed in copy numbers of the target (*hmg* and MON810 copies) in the non-diluted sample or as %MON810.

Cv: coefficient of variability between the four measurements. Expressed as a percentage of the average value.

Bias non-digested/digested: Bias of the ddPCR average values obtained on non-digested DNA in comparison with the average values obtained on digested DNA. Expressed as a percentage of the target value.
