# Supplementary material for: Quantitative Analysis of Food and Feed Samples with Droplet Digital PCR
Source: PLoS One. 2013 May 2;8(5):e62583. doi: 10.1371/journal.pone.0062583 (PMC3642186; doi:10.1371/journal.pone.0062583)
Supplement: Table S7 — False-positive rates observed with ddPCR. (DOC) [file pone.0062583.s008.doc]

Table S- : False-positive rates observed with ddPCR

|  | ***hmg*** | | | | **MON810** | | | |
| --- | --- | --- | --- | --- | --- | --- | --- | --- |
| Sample | Positive reactions | % positive reactions | Positive droplets | % positive droplets | Positive reactions | % positive reactions | Positive droplets | % positive droplets |
| G031/12 | 0/8* | 0% | 1/106,133 | 9e-4% | 0/8* | 0% | 1/106,133 | 9e-4% |
| G053/12 | n.a. | n.a. | n.a. | n.a. | 0/8 | 0% | 0/110,903 | 0% |
| TOTAL | 0/8* | 0% | 1/106,133 | 9e-4% | 0/16* | 0% | 1/217,036 | 5e-4% |

The false-positive rate for both targets can be calculated, based either on the number of false-positive reactions or on the number of false-positive droplets.

Positive reactions: number of positive reactions (at least 2 positive droplets)/number of accepted droplets

* One reaction resulted in one positive droplet and therefore was considered as negative.

n.a.: not applicable.
