# Supplementary material for: Quantitative Analysis of Food and Feed Samples with Droplet Digital PCR
Source: PLoS One. 2013 May 2;8(5):e62583. doi: 10.1371/journal.pone.0062583 (PMC3642186; doi:10.1371/journal.pone.0062583)
Supplement: Appendix S1 — Supporting material and methods. (PDF) [file pone.0062583.s009.pdf]

Supporting material and methods

Details on test material used in this study

See also Table S-1.

Certified reference materials (CRM)

MON-ØØ81Ø-6 (MON810) maize seed powder based CRMs purchased from the EU Joint Research Centre, IRMM (Institute for Reference Materials and Measurements, Geel, Belgium): ERM-BF413d (1.0% ±0.3% m/m and 0.57% ±0.17% cp/cp), ERM-BF413f (5.0% ±0.2% m/m), ERM-BF413ek (1.98% ± 0.15% m/m and 0.77% ± 0.08% cp/cp) and ERM-BF413gk (9.9% ± 0.5% m/m).

ERM-BF413gk is only certified for the mass/mass ratio. However, it belongs to the same series as ERM-BF413ek, meaning that they were both prepared with the same wild-type and MON810 maize powdered material [1]. Therefore, based on the correction factor between the mass/mass and the copy/copy certified values in the ERM-BF413ek CRM (factor 2.57), one can estimate the copy/copy MON810 content in ERM-BF413gk (3.85% ±0.2% cp/cp) from its mass/mass certified value. A similar evaluation can be applied for the copy/copy ratio in ERM-BF413f (2.85% ±0.1% cp/cp) issued from the same CRM series as ERM-BF413d [2], using the correction factor between the mass/mass and the copy/copy certified values in the ERM-BF413d CRM (factor 1.75).

Other test materials

Maize seed-powder flour samples: G0009/04 from the USDA-GIPSA proficiency program (January 2004, sample 4) (measured 0.29% ±0.13% cp/cp by qPCR, assigned value 0.3% m/m); G0180/07 from the USDA-GIPSA proficiency program (November 2007, sample 4) (measured 0.04% ±0.02% cp/cp by qPCR, assigned less than 0.1% m/m); G211/10 from the ILC-CRL-GMFF proficiency program (EURL-CT-02/10, sample level 1) (measured 0.50% ±0.15% cp/cp by qPCR, assigned value 0.45% ± 0.098% cp/cp) and G212/10 from the ILC-CRL-GMFF proficiency program (EURL-CT-02/10, sample level 2) (measured 2.30% ±0.7% cp/cp by qPCR, assigned value 2.10% ± 0.35% cp/cp).

Wheat seed-powder flour samples with maize contamination from the GEMMA proficiency test program were tested (G0147/08, measured 29.6% ±8.9% cp/cp with qPCR, no assigned value).

Two samples from routine GMO testing were included in the evaluation: corn flakes (G231/11, 2.64% ±0.8% cp/cp), and feed containing maize (G254/11, 3.82% ±1.1% cp/cp).

For specificity studies, two samples were used: the feed mix sample G053/12, containing high levels of MON40-3-2 soybean (MON-Ø4Ø32-6), and wild-type maize (but no MON810). The milk sample G031/12 contains no maize DNA but the extract contains a large amount of DNA, as shown by the low

quantification cycle (Cq) value obtained with a qPCR assay targeting the 18S rRNA gene (Applied Biosystems, Foster City, CA).

Modified CTAB method

A CTAB method [3], with the following modified composition was used: 200 mg of starting material, 600 µL of added water, 1000 µL of CTAB extraction buffer, and 40 µL of each RNase and proteinase K.

Enzymatic restriction of genomic DNA

The effect of gDNA endonuclease restriction on ddPCR accuracy was evaluated.

For enzymatic digestion, approximately 50 ng of MON810 genomic DNA (gDNA) were used in a total reaction volume of 30 µL containing the NEB4 10x restriction buffer, with 40 units of *Taq*I (New England Biolabs GmbH, Frankfurt am Main, Germany). The final volume was adjusted with nuclease- and protease-free water (Sigma-Aldrich Chemie GmbH, Munich, Germany) and incubated for 2 h at 65 °C. The *Taq*I enzyme was inactivated by incubating at 80 °C for 10 min. 6 µL of digested gDNA were analyzed on a 1% agarose gel to confirm complete digestion.

Two series (non-digested, and digested with *Taq*I) of four dilutions of DNA extracted from the ERM-BF413ek CRM were tested with the duplex ddPCR assay. The two series showed similar results in terms of linearity for the targets – 0.9995 and 0.9982 for the *hmg* and MON810 targets in the non-digested (native) gDNA series, and 0.9994 and 0.9966 for the *hmg* and MON810 targets in the *Taq*I digested gDNA series (data not shown). Estimates of copy number differed slightly: estimates with digested gDNA were about 30% lower than those with the native DNA. Moreover, copy number determination is more variable between dilution levels with the digested than with the native DNA (Table S-4). However, the measured MON810 contents were very similar in both series, through all the dilution series (bias = -0.28%).

Details on qPCR reactions and data analysis

Singleplex qPCR reaction mixes comprised 10 µL of 2× TaqMan® Universal mastermix (Applied Biosystems, Foster City, CA), the relevant primers at final concentrations of 300 nM, the relevant probe at a final concentration of 180 nM, and 4 µL DNA template (Table S-2). Duplex qPCR reaction mixes comprising 10 µL of 2× TaqMan® Universal mastermix (Applied Biosystems, CA, USA), the *hmg* primers at final concentrations ranging from 150 nM to 300 nM, the MON810 primers at final concentrations ranging from 300 nM to 600 nM (ratio *hmg*/MON810 primer from 1 to 4), the *hmg* probe at a final concentration ranging from 90 nM to 180 nM, the MON810 probe at a final concentration ranging from 180 nM to 360 nM (ratio *hmg*/MON810 primer from 1 to 4), and 4 µL DNA template. The final volume of all qPCR reactions was 20 µL. Primers and probes were purchased at Eurofins MWG Operon (Ebersberg, Germany).

All qPCR reactions were performed on a 7900HT Fast Real-Time PCR System (Applied BioSystems, Foster City, CA) with the following thermal cycling conditions: a 2 min uracil-N-glycosylase (AmpErase®) step at 50 °C, 10 min activation at 95 °C followed by 45 cycles of a two-step thermal profile comprising 15 s denaturation at 95 °C, and 60 s annealing/extension at 60 °C. SDS 2.3 software (Applied Biosystems, Foster City, CA) was used for data acquisition and analysis after manual adjustment of the baseline and fluorescence threshold. After being exported, further data analysis was performed in a Microsoft Excel spreadsheet (Microsoft, Redmond, WA).

Details on droplet digital PCR reactions and data analysis

Droplets were generated in 8-well cartridges, using the QX100 droplet generator (Bio-Rad, Pleasanton, CA) as described. Water-in-oil emulsions were transferred to a 96-well plate and amplified in a conventional calibrated GeneAmp 9700 PCR cycler (Applied BioSystems, Foster City, CA). Thermal cycling conditions were: 10 min denaturation at 95 °C, followed by 40 cycles of a two-step thermal profile comprising 15 s denaturation at 95 °C, and 60 s annealing/extension at 100% ramp rate at 60 °C. After amplification, products were denatured at 98 °C for 10 minutes, then cooled to 12 °C. Plates were then transferred to the QX100 droplet reader (Bio-Rad, Pleasanton, CA). Data acquisition and analysis was performed using QuantaSoft (Bio-Rad, Pleasanton, CA).

Positive droplets, containing amplification products, were discriminated from negative droplets without amplification products by applying a fluorescence amplitude threshold in QuantaSoft software (Bio-Rad, Pleasanton, CA). The threshold was set manually at the lowest point of the negative droplet cluster, as visualized using both the fluorescence amplitude vs. event number and the histogram of events vs. amplitude data streams, on each of the FAM and VIC channels.

Data generated by the QX100 droplet reader were rejected from subsequent analysis if a clog was detected by the Quantasoft software or if a low number (<10,000) of droplets was measured per 20 µL PCR. After being exported, the data were further analyzed in Microsoft Excel spreadsheets.

The percentage of positive droplets for a given target was calculated as the percentage of droplets showing the signal for the amplicon in the total number of analyzed droplets.

$$\%\ positive\ droplets\ = (droplet\ with\ positive\ signal\ \div total\ number\ of\ analyzed\ droplets)\ \times 100$$

Specificity

In the DNA extracted from a milk sample (G031/12), one reaction gave a unique positive droplet for hmg and another resulted in one positive droplet for MON810. Following the criterion set for positive reaction (at least two positive droplets), these reactions should be considered as negative. In the DNA extracted from the feed sample G053/12, no positive droplet was observed for the MON810 target. Taken together, the results suggest a false-negative rate of 0% (0/8 reactions) for the hmg target and 0% (0/16 reactions) for the MON810 target. However, if calculated on the basis of the droplet number, the

false-positive rate for ddPCR drops to 9.10^{-4} % (1 positive out of 106,133 analyzed) and 5.10^{-4} % (1 positive out of 217,036 analyzed) for hmg and MON810, respectively (Table S-7).

References

1. Broothaerts W, Contreras M, Corbisier P, Jeynov B, Kortekaas AM et al. (2009) Certification of Reference Materials of Maize Seed Powder containing Genetically Modified MON 810 Maize, ERM®-BF413k (ERM®-BF413ak, ERM®-BF413ck, ERM®-BF413ek, ERM®-BF413gk). Available: https://irmm.jrc.ec.europa.eu/html/reference_materials_catalogue/catalogue/attachements/ERM-BF413k_report.pdf. Accessed 24-9-2012.

2. Trapmann S, Le Guern L, Prokisch J, Robouch P, Kramer GN et al. (2001) The Certification of Reference Materials of Dry Mixed Maize Powder with different Mass Fractions of MON 810 Maize - Certified Reference Materials IRMM-413. Available: https://irmm.jrc.ec.europa.eu/refmat_pdf/ERM-BF413_report.pdf. Accessed 24-9-2012.

3. Somma M (2006) Extraction and purification of DNA. In: Querci M, Jermini.M., Van den Eede G, editors. Training Course on the Analysis of Food Samples for the Presence of Genetically Modified Organisms - User Manual. Luxembourg: Office for Official Publications of the European Communities.
