# Supplementary material for: Quantitative Analysis of Food and Feed Samples with Droplet Digital PCR
Source: PLoS One. 2013 May 2;8(5):e62583. doi: 10.1371/journal.pone.0062583 (PMC3642186; doi:10.1371/journal.pone.0062583)
Supplement: Appendix S3 — MIQE checklist. (PDF) [file pone.0062583.s011.pdf]

MIQE checklist

Item to check	Importance	Included?	Comments
**Experimental design**			
Definition of experimental and control groups	E	Yes	
Number within each group	E	Yes	
Assay carried out by the core or investigator’s laboratory?	D	Yes	Investigator's lab
Acknowledgment of authors’ contributions	D	Yes	
**Sample**			
Description	E	Yes	
Volume/mass of sample processed	D	Yes	
Microdissection or macrodissection	E	No	n.a.
Processing procedure	E	No	n.a.
If frozen, how and how quickly?	E	No	n.a.
If fixed, with what and how quickly?	E	No	n.a.
Sample storage conditions and duration (especially for FFPEb samples)	E	Yes	All samples stored at -20°C
**Nucleic acid extraction**			
Procedure and/or instrumentation	E	Yes	
Name of kit and details of any modifications	E	Yes	
Source of additional reagents used	D	Yes	
Details of DNase or RNase treatment	E	No	n.a.
Contamination assessment (DNA or RNA)	E	No	n.a.
Nucleic acid quantification	E	Yes	
Instrument and method	E	Yes	
Purity (A260/A280)	D	No	n.a.
Yield	D	No	n.a.
RNA integrity: method/instrument	E	No	n.a.
RIN/RQI or Cq of 3. and 5. transcripts	E	No	n.a.
Electrophoresis traces	D	No	n.a.
Inhibition testing (Cq dilutions, spike, or other)	E	Yes	Cq dilutions
**Reverse transcription**			
Complete reaction conditions	E	No	n.a.
Amount of RNA and reaction volume	E	No	n.a.
Priming oligonucleotide (if using GSP) and concentration	E	No	n.a.
Reverse transcriptase and concentration	E	No	n.a.
Temperature and time	E	No	n.a.
Manufacturer of reagents and catalogue numbers	D	No	n.a.
Cqs with and without reverse transcription	D	No	n.a.
Storage conditions of cDNA	D	No	n.a.
**qPCR target information**			
Gene symbol	E	Yes	HMGA1. n.a. for MON810 target
Sequence accession number	E	Yes	AJ131373 (*hmg*), AF434709 (MON810)
Location of amplicon	D	No	719 - 797 (intron 4 on acc. AJ131373 -hmg), 767 - 868 (junction region on acc. AF434709 -MON810)
Amplicon length	E	Yes	79bp (*hmg*), 92 bp (MON810)
In silico specificity screen (BLAST, and so on)	E	Yes	Blastn: amplicon aligns only on Zea mays hmgA gene (AJ131373) and a reference plasmid (JX434027) (*hmg assay*), and on transgenic maize (acc. No JQ406879 and AF434709) (MON810 assay)
Pseudogenes, retropseudogenes, or other homologs?	D	Yes	not found
Sequence alignment	D	No	
Secondary structure analysis of amplicon	D	Yes	mfold analysis of target sequence (50°C, 2.5 mM Mg2+, 50 mM Na+): 7 structures with deltaG from = -0.88 to -0.07 kcal/mol (for *hmg*) and two structures with deltaG from = -3.89 to -3.63 kcal/mol (for MON810)
Location of each primer by exon or intron (if applicable)	E	Yes	Both primers on intron 4 of acc. AJ131373 (*hmg*)
What splice variants are targeted?	E	No	n.a.
**qPCR oligonucleotides**			
Primer sequences	E	Yes	
RTPrimerDB identification number	D	No	n.a.
Probe sequences	D	Yes	
Location and identity of any modifications	E	Yes	Modification of the quenchers (BHQ1)
Manufacturer of oligonucleotides	D	Yes	Eurofins MWG Operon
Purification method	D	Yes	Desalting (HPSF®)
**qPCR protocol**			
Complete reaction conditions	E	Yes	
Reaction volume and amount of cDNA/DNA	E	Yes	
Primer, (probe), Mg2 &#1;, and dNTP concentrations	E	Yes	
Polymerase identity and concentration	E	Yes	
Buffer/kit identity and manufacturer	E	Yes	Bio-Rad (Hercules, CA), cat#186-3010
Exact chemical composition of the buffer	D	No	Not disclosed by the manufacter
Additives (SYBR Green I, DMSO, and so forth)	E	No	n.a.
Manufacturer of plates/tubes and catalog number	D	Yes	Eppendorf (Hamburg, Germany), cat. #0030 128.605
Complete thermocycling parameters	E	Yes	
Reaction setup (manual/robotic)	D	Yes	Manual
Manufacturer of qPCR instrument	E	Yes	
**qPCR validation**			
Evidence of optimization (from gradients)	D	No	n.a.
Specificity (gel, sequence, melt, or digest)	E	No	n.a. (done in ring trial for qPCR assays)
For SYBR Green I, Cq of the NTC	E	No	n.a.
Calibration curves with slope and y intercept	E	No	n.a. for ddPCR (no standard curve).
PCR efficiency calculated from slope	E	No	n.a. for ddPCR (end-point, no standard curve needed)
CIs for PCR efficiency or SE	D	No	n.a. for ddPCR (end-point).
r2 of calibration curve	E	Yes	0.9990 (hmg) and 0.993 (MON810) in linear range
Linear dynamic range	E	Yes	From 5 to 118,000 copies (*hmg*), from 18 to 4340 copies (highest point measured for MON810)
Cq variation at LOD	E	No	n.a. for ddPCR (end-point, droplet count)
CIs throughout range	D	No	
Evidence for LOD	E	Yes	At least two positive droplets detected in 5 x 20,000 droplets. LOD = 5 copies (*hmg*) and 6 copies (MON810)
If multiplex, efficiency and LOD of each assay	E	Yes	LOD of each assay determined. Efficiency not determined (n.a. for ddPCR)
**Data analysis**			
qPCR analysis program (source, version)	E	Yes	
Method of Cq determination	E	No	n.a. Based on droplet count
Outlier identification and disposition	E	Yes	
Results for NTCs	E	Yes	0% (n = 12)
Justification of number and choice of reference genes	E	Yes	Same as in validated qPCR assays
Description of normalization method	E	No	n.a.

Number and concordance of biological replicates	D	Yes	cv analysis of the replicates
Number and stage (reverse transcription or qPCR) of technical replicates	E	Yes	
Repeatability (intraassay variation)	E	Yes	
Reproducibility (interassay variation, CV)	D	No	n.a. (done in only one laboratory and istrument)
Power analysis	D	No	
Statistical methods for results significance	E	Yes	Via cv analysis.
Software (source, version)	E	Yes	Excel spreadsheet
Cq or raw data submission with RDML	D	No	

Checklist from Bustin *et al.* Clinical Chemistry 55:4 611–622 (2009) showing (E) essential and (D) desirable information to be included in research reports using qPCR.

* Information not provided in this work but reference is made to original publication for the validation of the qPCR assays. http://gmo-crl.jrc.ec.europa.eu/summaries/Mon810_validation_report.pdf
n.a. : not applicable to the study.
